# Modeling the role of respiratory droplets in Covid-19 type pandemics

**DOI:** 10.1063/5.0015984

**Published:** 2020-06-01

**Authors:** Swetaprovo Chaudhuri, Saptarshi Basu, Prasenjit Kabi, Vishnu R. Unni, Abhishek Saha

**Affiliations:** 1 Institute for Aerospace Studies, University of Toronto, Toronto, Ontario M3H 5T6, Canada; 2 Department of Mechanical Engineering, Indian Institute of Science, Bengaluru, Karnataka 560012, India; 3 Department of Mechanical and Aerospace Engineering, University of California San Diego, La Jolla, California 92093, USA

## Abstract

In this paper, we develop a first principles model that connects respiratory droplet physics with the evolution of a pandemic such as the ongoing Covid-19. The model has two parts. First, we model the growth rate of the infected population based on a reaction mechanism. The advantage of modeling the pandemic using the reaction mechanism is that the rate constants have sound physical interpretation. The infection rate constant is derived using collision rate theory and shown to be a function of the respiratory droplet lifetime. In the second part, we have emulated the respiratory droplets responsible for disease transmission as salt solution droplets and computed their evaporation time, accounting for droplet cooling, heat and mass transfer, and finally, crystallization of the dissolved salt. The model output favourably compares with the experimentally obtained evaporation characteristics of levitated droplets of pure water and salt solution, respectively, ensuring fidelity of the model. The droplet evaporation/desiccation time is, indeed, dependent on ambient temperature and is also a strong function of relative humidity. The multi-scale model thus developed and the firm theoretical underpinning that connects the two scales—macro-scale pandemic dynamics and micro-scale droplet physics—thus could emerge as a powerful tool in elucidating the role of environmental factors on infection spread through respiratory droplets.

## I. INTRODUCTION

It has been well established that the SARS-CoV-2 virus responsible for the Covid-19 pandemic transmits via respiratory droplets that are exhaled during talking, coughing, or sneezing.^1^ Each act of expiration corresponds to different droplet sizes and myriad trajectories for the droplets embedded in the corresponding jets. Wells^2,3^ was the first to investigate the role of respiratory droplets in respiratory disease transmission. Expelled respiratory droplets from an average human being contain dissolved salt with a mass fraction of about 0.01 as well as various proteins and pathogens in varying concentrations.^4,5^ In this paper, to model the outbreaks, we extensively use the evaporation and settling dynamics of NaCl–water droplets as a surrogate model of the infectious droplets. Stilianakis and Drossinos^6,7^ included respiratory droplets in their epidemiological models. However, they neglected the droplet evaporation dynamics and assumed that characteristic post-evaporation droplet diameters are half of the pre-evaporation droplet diameters based on Nicas *et al.*^8^ In the context of the present Covid-19 pandemic, while the role of droplet nuclei and corresponding “aerosol transmission” route are not clear,^1^ it is widely accepted that respiratory droplets are definitely a dominant vector in transmitting the SARS-CoV-2 virus. This merits a detailed investigation of the evaporation dynamics of respiratory droplets and development of a pandemic model that is explicitly dependent on the respiratory droplet characteristics. As such, the evaporation mechanism of respiratory droplets are laced with complexities stemming from droplet aerodynamics, initial droplet cooling, heat transfer, mass transfer of the solvent and solute, respectively, and finally, crystallization of the solute—a phenomenon known as efflorescence. All these are strongly affected by ambient conditions in which the droplet evaporates. These urgently necessitate a model based on first principles, which connects the detailed evaporation dynamics of respiratory droplets with the pandemic evolution equations. In this paper, a model for the infection rate constant based on collision theory incorporates the evaporation physics of respiratory droplets, *ab initio*.

The droplet evaporation model thus developed is first validated with new experimental results obtained from droplets observed to evaporate in an acoustic levitator. While very interesting insights can be obtained from sessile droplet evaporation,^9–13^ after an expiratory event, the floating droplet evaporates in the absence of surface contact. Thus, the levitated droplets are similar to the droplets in atmosphere^14–16^ compared to their sessile counterpart. Furthermore, the desiccation dynamics necessitates a contact-less environment for the droplet. Alongside a droplet evaporation model, a chemical kinetics based reaction mechanism model is developed with final rate equations similar to that yielded by the SIR (Susceptible, Infectious, Recovered) model.^17^ In general, the resemblance of the equations modeling kinetics to those of population dynamics is well known. However, the rigorous framework (analytical as well as computational) of chemical reaction mechanisms that can at present handle few thousands of species and tens of thousands of elementary reactions seems particularly attractive.^18^ This could be utilized toward adding further granularity in the pandemic model, if required large mechanisms can be reduced systematically with mechanism reduction techniques.^19^ Furthermore, it can be integrated into advection-diffusion-reaction equations, and their moments could be solved using appropriate moment-closure methods.^20,21^ However, for any reaction mechanism, the key inputs are the parameters for the reaction rate constant. In our case, one rate constant is shown to be a strong function of the droplet lifetime. Therefore, next, the droplet lifetime is evaluated over a wide range of conditions relevant to the ongoing Covid-19 pandemic, and the growth rate exponents (eigenvalues) are presented. The results do not suggest that factors not considered in this paper play a secondary role in determining the outbreak spread. Rather, this paper aims to establish the mathematical connection between the pandemic and the respiratory droplet dynamics using a well defined framework rooted in physical sciences. This paper is arranged as follows: first, we provide details of the experiments used to obtain the evaporation characteristics of the water and salt solution droplets. This is followed by the reaction mechanism model that yields the equations for the growth rate and the infection rate constant of the outbreaks. This infection rate constant provides the connection and motivation for modeling the droplet evaporation time scales. Next, to evaluate the rate constant, detailed modeling of the droplet evaporation is presented. This is followed by results and discussions. Finally, we summarize the approach and findings in Sec. VII.

## II. EXPERIMENTS

**FIG. 1.**
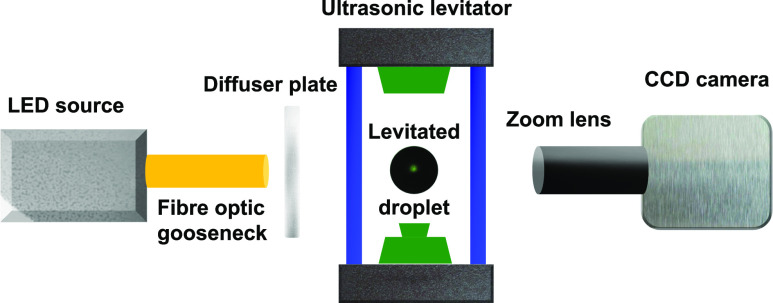
Experimental setup showing the acoustic levitation of a droplet illuminated by a cold LED source. A diffuser plate is used for uniform imaging of the droplet. A CCD camera fitted with the zoom lens assembly is used for illumination. The schematic is not to scale.

The experiments with isolated evaporating droplets were conducted in a contact-less environment of an ultrasonic levitator (tec5) to discount boundary effects, generally present in suspended, pendant, or sessile droplet setups.^22,23^ The experimental setup with the diagnostics is shown in Fig. 1. A droplet was generated and positioned near one of the stable nodes of the levitator by using a micropipette. The levitated droplet was allowed to evaporate in the ambient condition of the laboratory at 30 °C and at about 50% relative humidity (RH). The transient dynamics of evaporation and precipitation of the evaporating droplet was captured with the shadowgraphy technique using a combination of a CCD camera (NR3S1, IDT Vision) fitted with a Navitar zoom lens assembly (6.5× lens and 5× extension tube) and a backlit-illumination by a cold LED light source (SL150, Karl Storz).

A set of ten images at a burst speed of 30 fps is acquired every 2 s for the entire duration of the droplet lifetime. The spatial resolution of the images was ≈1 *μ*m/pixel. The temporal evolution of the diameter of the evaporating droplet was extracted from the images using the “Analyze Particles” plugin in ImageJ (open source platform for image processing). The final precipitate was carefully collected on carbon tape and observed in the dark-field mode under a reflecting microscope (Olympus BX-51). A range of initial droplet diameters varying from 300 *µ*m to 1000 *µ*m were investigated in experiments.

## III. A REACTION MECHANISM TO MODEL THE PANDEMIC

**FIG. 2.**
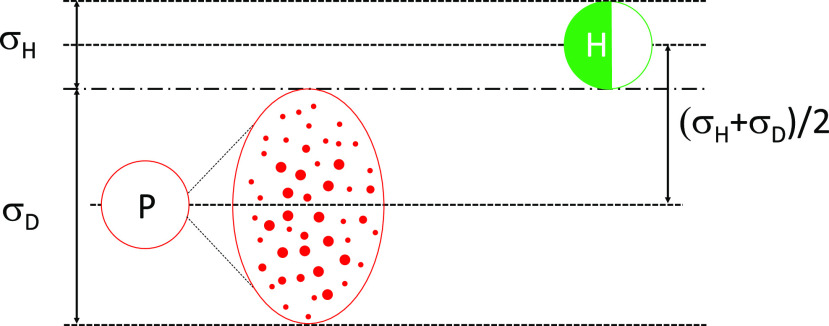
A schematic of the collision rate model for the infection to occur. Infected person *P* ejects a cloud of infectious droplets *D* denoted by small red dots, and the cloud approaches a healthy person *H* with a relative velocity ${\vec{V}}_{DH}$ to infect them. The figure also shows the collision volume swept by the droplet cloud *D* and *H* with their respective effective diameters.

In this section, we model the infection spread rate using the collision theory of reaction rates, well known in chemical kinetics.^18^ The connection between droplets and the outbreak will be established later. In this model, we adopt the following nomenclature: *P* represents a Covid-19 positive person infecting a healthy person(s) susceptible to infection. The healthy person is denoted by *H* (who is initially Covid-19 negative), and *R* represents a person who has recovered from Covid-19 infection and hence assumed to be immune from further infection, while *X* represents a person who dies due to Covid-19 infection. We consider one-dimensional head on collisions, and the schematic of a collision volume is shown in Fig. 2. Here, one healthy person denoted by *H* with the effective diameter *σ*_*H*_ is approached by a Covid-19 positive person *P* of the same effective diameter with an average relative velocity ${\vec{V}}_{DH}$. *σ*_*H*_ can be considered as the diameter of the hemispherical volume of air that is drawn by *H* during each act of inhalation, which comes out to be approximately 0.124 m.

It is widely believed that Covid-19 spreads by respiratory droplets^24^ resulting from breathing, coughing, sneezing, or talking. Thus, we assume that a volume in front of *P* is surrounded by a cloud of infectious droplets exhaled by *P*. The droplet cloud is denoted by *D*, and the maximum cloud diameter is given by *σ*_*D*_. Clearly, *σ*_*D*_ should be determined by the smaller of evaporation or settling time of the droplets ejected by *P*, the horizontal component of the velocity with which the droplets traverse, as well as the dispersion characteristics. In each such cloud, we assume that there are numerous droplets containing the active Covid-19 virus. The velocity of this droplet cloud relative to *H* is given by ${\vec{V}}_{DH}$. In such a scenario, we assume that in a unit volume, there are *n*_*P*_ infected persons and *n*_*H*_ healthy persons. For a collision to be possible, the maximum separation distance between the centers of *D* (the droplet cloud) and *H* is given by

$$\sigma_{DH}=\left(\sigma_{D}+\sigma_{H}\right)/2.$$ (1)

The collision volume—the volume of the cylinder within which a collision between the droplet cloud of *P* and air collection volume of *H* should lie for the collision to occur in a unit time—is given by $\pi \sigma_{DH}^{2}V_{DH}$. Thus, the number of collisions between *H* and the droplet cloud *D* of *P*, per unit time per unit volume, that will trigger infections, is given by

$$Z_{DH}=\pi \sigma_{DH}^{2}V_{DH}n_{P}n_{H},$$ (2)

where *n*_*P*_ and *n*_*H*_ represent the number of *P* and *H*, respectively. Now, given that each collision between *P* (basically, its droplet cloud *D*) and *H* results in conversion of the healthy individual to the infected individual, we can write

$$\frac{dn_{H}}{dt}=-Z_{DH}.$$ (3)

Now, we can define [*P*] = *n*_*P*_/*n*_*total*_ and [*H*] = *n*_*H*_/*n*_*total*_, whereas *n*_*total*_ is the total number of people those who are capable of transmitting the infection, as well as accepting the infection per unit volume, in that given volume. This implies

$$\omega =-\frac{d\left[H\right]}{dt}=n_{total}\pi \sigma_{DH}^{2}V_{DH}\left[P\right]\left[H\right]=k\left[P\right]\left[H\right],$$ (4)

where

$$k=n_{total}\pi \sigma_{DH}^{2}V_{DH}.$$ (5)

Here, *ω* is the reaction rate. Furthermore, if we assume that the mortality rate is about 3% for the ongoing Covid-19 pandemic, we can convert the kinetics of infection spread to a complete reaction mechanism given by the following:

$$P+H\overset{k_{1}}{\to }P+P^{*}\;\left[R1\right],$$

$$\;P^{*}\overset{k_{2}}{\to }P\;\left[R2\right],$$

$$\;P\overset{k_{3}}{\to }0.97R+0.03X\;\left[R3\right].$$

It is to be recognized that *H* does not become *P* immediately on contact with the droplet cloud. The virus must proliferate for a finite time after contact to render a person infectious. A person who has just come in contact with the virus and does not have the capability to infect others yet is denoted by P^*^. *k*_1_, *k*_2_, and *k*_3_ are the rate constants of reactions [R1], [R2], and [R3], respectively. All rate constants must have dimensions of [*T*]^−1^ (inverse of time). Clearly, *k*_1_ > *k*_3_ for the rapid outbreak to occur. It is to be recognized that this framework implies that *k*_1_, the rate constant of the second order elementary reaction [*R*1] resulting from collisions between the droplet cloud from an infectious individual and healthy individual, is purely controlled by physical effects. The rate constants *k*_2_ and *k*_3_ of the other two first order elementary reactions [*R*2] and [*R*3] are essentially decay rates emerging from the time by which the respective concentrations reach *e*^−1^ levels of the initial concentration for the respective reactions. Thus, *k*_2_ and *k*_3_ are purely determined by interaction between the virus and the human body. We know that the approximate recovery time from the Covid-19 disease is about 14 days. Thus, we can assume *k*_3_ = 1/14 day^−1^. We also assume the latency period (not incubation period) to be 1 day; hence, *k*_2_ = 1 day^−1^. Given the importance of *k*_1_ in determining the outbreak characteristics, we will refer to *k*_1_ as the infection rate constant. The major contribution of this work is imparting a rigorous physical interpretation to *k*_1_ and calculating it.

Using Eq. (4), we can write the system of ODEs for *d*[*P*]/*dt* and *d*[P^*^]/*dt* as

$$\left[\begin{array}{c} \frac{d\left[P\right]}{dt}\\ \frac{d\left[P^{*}\right]}{dt} \end{array}\right]=\left[\begin{array}{cc} -k_{3} & \;k_{2}\\ k_{1}\left[H\right] & \;-k_{2} \end{array}\right]\left[\begin{array}{c} \left[P\right]\\ \left[P^{*}\right] \end{array}\right].$$ (6)

In this paper, we are interested in modeling the initial phases of the outbreaks where [*H*] ≫ [*P*]. Hence, we can safely assume [*H*] ≈ [*H*]_0_, i.e., the concentration of healthy people remains approximately constant during the early phase of the outbreak and is equal to the initial concentration, which is very close to unity at *t* = 0, i.e., at the onset of the outbreak. The time of the beginning of the outbreak denoted by *t* = 0 for a particular location can be assumed to be the day when the number of Covid-19 positive persons equaled 10. [*P*]_0_ is [*P*] at *t* = 0. Then, [*P*] can be solved as an eigenvalue problem and is given by

$$\left[P\right]={\left[P\right]}_{0}\left(C_{1}e^{\lambda_{1}t}+C_{2}e^{\lambda_{2}t}\right).$$ (7)

*C*_1_ and *C*_2_ are constants to be determined from the eigenvectors and the initial conditions [*P*]_0_ and ${\left[P^{*}\right]}_{0}$. *λ*_1,2_ are the eigenvalues. These can be termed growth parameters and are given by

$$\lambda_{1,2}=\left\{-\left(k_{3}+k_{2}\right)\pm \sqrt{{\left(k_{3}+k_{2}\right)}^{2}-4\left(k_{2}k_{3}-k_{1}k_{2}\right)}\right\}/2.$$ (8)

By Eq. (5), $k_{1}=n_{total}\pi \sigma_{DH}^{2}V_{DH}$. As mentioned before, *k*_2_ = 1 day^−1^ and *k*_3_ = 1/14 day^−1^, which yields $\lambda_{1,2}=-0.5357\pm \sqrt{0.2156+k_{1}}$. If *k*_2_ → ∞, i.e., a healthy person becomes infectious immediately on contact with an infectious person, *λ*_1_ → *k*_1_ − *k*_3_.

Clearly, this model does not yet account for the preventive measures such as “social distancing,” “quarantining” after contact tracing, and population wide usage of masks. We will call this “social enforcement.” However, it can be included by accounting for the time variation in [*H*]. Social enforcement measures reduce the concentration of healthy, susceptible individuals from [*H*_0_] to [*H*_*SE*_] where the concentration of healthy population susceptible to infection after implementing strict social distancing (at time *t* = *t*_*SE*_) [*H*_*SE*_] < [*H*_0_]. In the case of social enforcement, [*P*] will be given by

$$\;\left[P\right]=\left\{\begin{array}{cc} {\left[P\right]}_{0}\left(C_{1}e^{\lambda_{1}t}+C_{2}e^{\lambda_{2}t}\right),\; & 0<t<t_{SE}\\ {\left[P\right]}_{SE}\left(C_{1}e^{\lambda_{1}\left(t-t_{SE}\right)}+C_{2}e^{\lambda_{2}\left(t-t_{SE}\right)}\right),\; & t\geq t_{SE} \end{array}\right..$$ (9)

Here, [*P*] = [*P*]_*SE*_ at *t* = *t*_*SE*_ and *λ*_1,*SE*_, *λ*_2,*SE*_ are the eigenvalues from Eq. (6) with [*H*] = [*H*_*SE*_]. *k*_1_, the infection rate constant, remains to be completely determined. It is to be recognized that two of the key inputs of *k*_1_ are *σ*_*DH*_ and *V*_*DH*_ since $k_{1}\propto V_{DH}\sigma_{DH}^{2}$ by Eq. (5). As already mentioned, *σ*_*H*_ is the diameter of the hemisphere from which breathable air is inhaled. *σ*_*D*_ is the diameter of the droplet cloud. The aerodynamics of the respiratory droplets needs to be analyzed to evaluate these quantities.

## IV. MODELING AERODYNAMICS OF RESPIRATORY DROPLETS

The droplets ejected during respiratory events, such as sneezing and coughing, co-follow the volume of air exhaled during the event. Studies have confirmed that due to entrainment, the exhaled air volume grows in diameter, while its kinetic energy decays with time. Specifically, Bourouiba *et al.*^16^ showed that initially, for a short duration, the droplets evolve inside a turbulent jet, while in later stages, the jet transitions to a puff. Recognizing that the ejected droplets during the respiratory event is surrounded by this dynamically evolving air volume and that the motion of the droplets will be strongly coupled due to the aerodynamic drag, we first model the surrounding air in two parts using the analytical results of the turbulent jet and puff, respectively. The axial location, axial velocity, and radial spread of a transient turbulent jet and puff can be expressed, respectively, as^25,26^

$$\begin{array}{cc} x_{j}\left(t\right) & ={\left(\frac{12}{K}\right)}^{1/2}{\left(U_{j,0}R_{j,0}\right)}^{1/2}t^{1/2},\\ U_{j}\left(t\right) & =\frac{3U_{j,0}R_{j,0}}{Kx_{j}\left(t\right)},\\ R_{j}\left(t\right) & =R_{j,0}+\frac{x_{j}\left(t\right)-x_{j,0}}{5}, \end{array}$$ (10)

and

$$\begin{array}{cc} x_{pf}\left(t\right) & =\left(\frac{3m}{a}\right)R_{pf}\left(t\right),\\ U_{pf}\left(t\right) & =U_{pf,0}{\left(\frac{3mR_{pf,0}}{4aU_{pf,0}t}\right)}^{3/4},\\ R_{pf}\left(t\right) & =R_{pf,0}{\left(\frac{4aU_{pf,0}t}{3mR_{pf,0}}\right)}^{1/4}, \end{array}$$ (11)

where subscripts *j* and *pf* denote the jet and puff, respectively. *R*_0_ and *U*_0_ are the radius and axial velocities at a distance *x*_0_. *K* is a characteristic constant for the turbulent jet and is reported to be 0.457.^25^ At the inception of the respiratory event (*t* = 0), the jet is assumed to have a velocity *U*_*j*,0_ = 10 m/s and a radius *R*_*j*,0_ = 14 mm—the average radius of human mouth. The characteristic constants for a puff are *a* ≈ 2.25 and *m* = (*x*_*p*,0_*a*)/(3*R*_*p*,0_).^26^ Since the continuous ejection of air from the mouth lasts only for the duration of a single respiratory event, the jet behavior persists only for this period and beyond which the puff behavior is observed. The average duration of such events is roughly 1 s.^27^ Hence, the velocity and the radial spread of the air surrounding the exhaled droplets will be

$$\begin{array}{c} U_{g}=\left\{\begin{array}{cc} U_{j}\left(t\right),\; & t\leq 1s\\ U_{pf}\left(t\right),\; & t>1s, \end{array}\right.\\ R_{g}=\left\{\begin{array}{cc} R_{j}\left(t\right),\; & t\leq 1s\\ R_{pf}\left(t\right),\; & t>1s. \end{array}\right. \end{array}$$ (12)

The horizontal displacement (*X*_*p*_) of the exhaled droplet and its instantaneous velocity (*U*_*p*_) due to the drag can be solved with^14^

$$\begin{array}{c} dX_{p}/dt=U_{p},\\ dU_{p}/dt=\left(\frac{3C_{D}\rho_{v}}{8R_{s}\rho_{l}}\right)|U_{g}-U_{p}|\left(U_{g}-U_{p}\right), \end{array}$$ (13)

where *R*_*s*_ is instantaneous radius of the droplet, *ρ*_*v*_ and *ρ*_*l*_ are gas phase and liquid phase densities, *μ*_*g*_ is gas phase dynamic viscosity, and *C*_*D*_ is the drag coefficient, which can be taken as 24/*Re*_*p*_ for the gas phase Reynolds number, *Re*_*p*_ = (2*ρ*_*v*_|*U*_*g*_ − *U*_*p*_|*R*_*s*_)/*μ*_*g*_ < 30.^14^ As it will be stated later, *Re*_*p*_ for the respiratory droplets were found to be mostly less than 0.1.

By solving Eqs. (10)–(13) over the droplet lifetime, *τ*, the axial distance traveled by the droplets, *X*_*p*_, can be evaluated. The average velocity of the droplet cloud relative to *P* is *V*_*D*,*P*_ = *X*_*p*_/*τ*. The diameter of the droplet cloud ejected by *P* can be approximated as twice the radial spread of the exhaled air, *σ*_*D*_ = 2*R*_*g*_(*t* = *τ*). It is to be recognized that while the above equations are analytically tractable, given the complexities of the associated turbulent jet/puff, a detailed description of the motion of the droplets necessitates time resolved Computational Fluid Dynamics (CFD) simulations in three dimensions. This has been recently reported in Ref. 28, which simulated dispersion of water droplets using a fully coupled Eulerian–Lagrangian technique including the wind effects. In this paper, we worked with salt solution droplets, accounting for salt crystallization, but did not include wind effects to retain analytical tractability. Nevertheless, the results presented in Subsection VI B are qualitatively consistent with the CFD results.

Due to evaporation or settling, the droplet is present only for a short time *τ* after it has been ejected. Therefore, the steady state *k*_1_ can be defined as

$$k_{1}=n_{total}\pi \sigma_{DH}^{2}V_{DH}\left(\tau /t_{c}\right).$$ (14)

Just like in collision theory, not all molecules are energetic enough to effect reactions; in our case, the droplet cloud is not always present. The last fraction (*τ*/*t*_*c*_) is the probability that the droplet cloud with the average diameter *σ*_*D*_ is present. *t*_*c*_ is the average time period between two vigorous expiratory events. *V*_*DH*_ = (*V*_*D*,*P*_ + *V*_*P*_) + *V*_*H*_. We can assume *V*_*P*_ = *V*_*H*_. It is thus apparent that *τ* appears in *σ*_*DH*_, *V*_*DH*_, and in the last fraction in Eq. (14), thereby emerging as a critical parameter of the entire pandemic dynamics. Hence, *τ* merits a detailed physical understanding. Given the composition of the respiratory droplets, modeling *τ* is highly non-trivial and is taken up in Sec. V.

## V. MODELING RESPIRATORY DROPLET EVAPORATION

It is well documented in the literature that an average human exhales droplets (consisting of water, salt, proteins, and virus/bacteria) in the range of 1 *µ*m–2000 *µ*m.^5,29,30^ In this section, we offer a detailed exposition of the evaporation dynamics of such droplets as ejected during the course of breathing, talking, sneezing, or coughing.

The small droplets (<2 *µ*m–3 *µ*m) have a very short evaporation timescale. This implies that these droplets evaporate quickly (<1 s) after being ejected. However, the same conclusion does not hold for slightly larger droplets ejected in the form of cloud (>5 *µ*m). These droplets exhibit longer evaporation time, leading to increased chances of transmission of the droplet laden viruses. In particular, when inhaled, these droplets enable quick and effective transport of the virus directly to the lungs airways causing a higher probability of infection. In general, the smaller droplets (<30 *µ*m) have low Stokes number, thereby allowing them to float in ambient air without the propensity to settle down. For larger droplets (>100 *µ*m), the settling timescale is very small (∼0.5 s). In effect, based on the diameter of the exhaled droplets, there are three distinct possibilities:

• Small droplets (<5 *µ*m) evaporate within a fraction of second.
• Large droplets (>100 *µ*m) settle within a small time frame (<0.5 s), limiting the radius of infection.
• Intermediate droplets (∼30 *µ*m) show the highest probability of infection due to a slightly longer evaporation lifetime and low Stokes number.

In this work, we particularly focus our attention to the modeling of droplets over a large range of diameters from 1 *µ*m to 100 *µ*m. Based on the available literature, we assume that the droplets exhaled during breathing are at an initial temperature of 30 °C.^31^ The ambient condition, however, vary strongly with geographical and seasonal changes, etc. Hence, in the following, we conduct a parametric study to determine the droplet lifetime across a large variation of temperature and relative humidity conditions. The droplet evaporation physics is complicated by the presence of non-volatile salts (predominantly NaCl) as present in our saliva.^4^ We would also look into simultaneous desiccation of the solvent and crystallization of such salts Subsections V A and V B. Once exhaled and encountering ambience, the droplet will evaporate as it undergoes simultaneous heat and mass transfer.

### A. Evaporation

For the modeling purpose, the exhaled droplets are assumed to evaporate in a quiescent environment at a fixed ambient temperature and relative humidity. In reality, during coughing, talking, or sneezing, the droplets are exhaled in a turbulent jet/puff.^16^ However, as shown in Eqs. (10) and (11), the puff rapidly decelerates due to entrainment and lack of sustained momentum source, rendering the average *V*_*D*,*P*_ to be less than 1% of the initial velocity. Furthermore, since the Prandtl number, defined as ratio of kinematic viscosity and thermal diffusivity, is approximately unity (*Pr* = *ν*/*α* ≈ 0.71) for air, we can safely assume that the temperature and relative humidity that the droplets in the puff experience are on average very close to that of the ambient. At the initial stages, the puff will indeed be slightly affected by buoyancy, which will influence droplet cooling and evaporation dynamics. Quantifying these effects accurately, merit separate studies, see for e.g., Ref. 32 for buoyant clouds. In a higher dimensional model, these could be incorporated. Nonetheless, the evaporation rate of the droplet is driven by the transport of water vapor from the droplet surface to the ambient far field. Assuming the quasi-steady state condition, the evaporation mass flux can be written as

$$\begin{array}{cc} {\dot{m}}_{1} & =-4\pi \rho_{v}D_{v}R_{s}log\left(1+B_{M}\right),\\ {\dot{m}}_{1} & =-4\pi \rho_{v}\alpha_{g}R_{s}log\left(1+B_{T}\right). \end{array}$$ (15)

Here, *ṁ*_1_ is the rate of change of the droplet water mass due to evaporation, *R*_*s*_ is the instantaneous droplet radius, *ρ*_*v*_ is the density of water vapor, *D*_*v*_ is the binary diffusivity of water vapor in air, and *α*_*g*_ is the thermal diffusivity of surrounding air. *B*_*M*_ = (*Y*_1,*s*_ − *Y*_1,∞_)/(1 − *Y*_1,*s*_) and *B*_*T*_ = *C*_*p*,*l*_(*T*_*s*_ − *T*_∞_)/*h*_*fg*_ are the Spalding mass transfer and heat transfer numbers, respectively. Here, *Y*_1_ is the mass fraction of water vapor, while subscripts *s* and ∞ denote the location at the droplet surface and at the far field, respectively. The numerical subscripts 1, 2, and 3 will denote water, air, and salt, respectively. *C*_*p*,*l*_ and *h*_*fg*_ are the specific heat and specific latent heat of vaporization of the droplet liquid. For the pure water droplet, the vapor at the droplet surface can be assumed to be at the saturated state. However, as indicated earlier, the exhaled droplets during talking, coughing, or sneezing are not necessarily pure water; rather, they contain plethora of dissolved substances.^5^ The existence of these dissolved non-volatile substances, henceforth denoted as solute, significantly affects the evaporation of these droplets by suppressing the vapor pressure at the droplet surface. The modified vapor pressure at the droplet surface for binary solution can be expressed by Raoult’s Law, *P*_*vap*_(*T*_*s*_, *χ*_1,*s*_) = *χ*_1,*s*_*P*_*sat*_(*T*_*s*_), where *χ*_1,*s*_ is the mole-fraction of the evaporating solvent (here water) at the droplet surface in the liquid phase^14^ and *χ*_1,*s*_ = 1 − *χ*_3,*s*_. The far field vapor concentration, on the other hand, is related to the relative humidity of the ambient. Considering the effects of Raoult’s law and relative humidity, the vapor concentrations at the droplet surface and at the far field can be expressed as

$$\begin{array}{cc} Y_{1,s} & =\frac{P_{vap}\left(T_{s},\chi_{1,s}\right)M_{1}}{P_{vap}\left(T_{s},\chi_{1,s}\right)M_{1}+\left(1-P_{vap}\left(T_{s},\chi_{1,s}\right)\right)M_{2}},\\ Y_{1,\infty } & =\frac{\left(RH\right)P_{sat}\left(T_{\infty }\right)M_{1}}{\left(RH\right)P_{sat}\left(T_{\infty }\right)M_{1}+\left(1-\left(RH\right)P_{sat}\left(T_{\infty }\right)\right)M_{2}}. \end{array}$$ (16)

$M_{1}$ and $M_{2}$ denote the molecular weights of water and air, respectively. For evaporation, the droplet requires latent heat, which is provided by the droplet’s internal energy and surrounding ambient. It has been verified that the thermal gradient in the liquid phase is rather small. Therefore, neglecting the internal thermal gradients, the energy balance is given by

$$mC_{p,l}\frac{\partial T_{s}}{\partial t}=-k_{g}A_{s}\frac{\partial T}{\partial r}{|}_{s}+{\dot{m}}_{1}h_{fg}-{\dot{m}}_{1}e_{l},$$ (17)

where *T*_*s*_ is instantaneous droplet temperature, $m=\left(4/3\right)\pi \rho_{l}R_{s}^{3}$ and $A_{s}=4\pi R_{s}^{2}$ are the instantaneous mass and surface area of the droplet, *ρ*_*l*_ and *e*_*l*_ are the density and specific internal energy of the binary mixture of salt (if present) and water, and *k*_*g*_ is the conductivity of gas surrounding the droplet. $\frac{\partial T}{\partial r}{|}_{s}$ is the thermal gradient at the droplet surface and can be approximated as (*T*_*s*_ − *T*_∞_)/*R*_*s*_, which is identical to convective heat transfer for a sphere with a Nusselt number of 2. As such, including aerodynamic effects, the Nusselt number is given by $Nu=2+0.6Re_{p}^{0.5}Pr^{0.3}$. The droplet Reynolds number, *Re*_*p*_, was observed to be mostly less than 0.1, and as such, the aerodynamic enhancement of the Nusselt number, i.e., the second term in the right-hand side, is ignored.

### B. Crystallization

Evaporative loss of water leads to an increase in the salt concentration in the droplet with time. As shown before, *P*_*vap*_(*T*_*s*_, *χ*_1,*s*_) is a function of the salt concentration in the droplet, which thus must be modeled using the species balance equation, as shown in the following equation:

$$\frac{dmY_{3}}{dt}+{\dot{m}}_{3,out}=0.$$ (18)

Here, *Y*_3_ is the dissolved solute (salt) mass fraction in the droplet. *ṁ*_3,*out*_, which represents the rate at which solute (salt) mass leaves the solution due to crystallization, is modeled below. Clearly, Eq. (18) shows that as water leaves the droplet, *Y*_3_ increases. When *Y*_3_ is sufficiently large such that the supersaturation ratio *S* = *Y*_3_/*Y*_3,*c*_ exceeds unity, crystallization begins. Here, we use *Y*_3,*c*_ = 0.393 based on the efflorescent concentration of 648 g/l reported for NaCl–water droplets in Ref. 33. The growth rate of the crystal could be modeled using a simplified rate equation from^34,35^

$$\frac{dl}{dt}={\left(S-1\right)}^{g_{cr}}C_{cr}e^{-E_{a}/RT_{s}}.$$ (19)

Here, *l* is the crystal length. Following Ref. 35, for NaCl, we find the constant *C*_*cr*_ = 1.14 × 10^4^ m/s, the activation energy *E*_*a*_ = 58 180 J/mol, and constant *g*_*cr*_ = 1. Using this, the rate of change of the crystal mass, which equals *ṁ*_3,*out*_, is given by^35^

$${\dot{m}}_{3,out}=\frac{dm_{3,crystal}}{dt}=6\rho_{s}{\left(2l\right)}^{2}\frac{dl}{dt}.$$ (20)

We note that while crystallization process could involve complex kinetics of solute, solvent, and ions; a well-studied^35^ single-step crystallization kinetics has been used here for tractability. It will be shown that this model is able to predict the experimentally studied droplet lifetime reasonably well.

The governing equations [Eqs. (15)–(20)] manifest that several physical mechanisms are coupled during the evaporation process. For *T*_*s*,0_ > *T*_∞_, the droplet undergoes rapid cooling from its initial value. The droplet temperature, however, should eventually reach a steady state limit (wet bulb). This limit is such that the droplet surface temperature will be lower than the ambient, implying a positive temperature gradient or heat input. The heat subsequently transferred from the ambient to the droplet surface after attaining the wet-bulb limit is used completely for evaporating the drop without any change in sensible enthalpy. For a droplet with pure water, i.e., no dissolved non-volatile content, the mole-fraction of the solvent at the surface remains constant at 100%, and at the limit of steady state, the droplet evaporation can be written in terms of the well-known *D*^2^ law,^14,18^

$$D_{s}^{2}\left(t\right)=D_{s,0}^{2}-K_{m}t,$$ (21)

where

$$K_{m}=8\left(\rho_{v}/\rho_{l}\right)D_{v}ln\left(1+B_{M}\right).$$ (22)

However, for a droplet with the binary solution, the evaporation becomes strongly dependent on the solvent (or solute) mole-fraction, which reduces (or increases) with evaporative mass loss. The transient analysis, thus, becomes critically important in determining the evolution of the droplet surface temperature and instantaneous droplet size. During evaporation, the mole-fraction of the solute increases and attains a critical super-saturation limit, which triggers precipitation. The precipitation and accompanied crystallization dynamics, essentially, reduce the solute mass dissolved the in liquid phase, leading to a momentary decrease in its mole-fraction. This, in turn, increases the evaporation rate as mandated by Raoult’s law, which subsequently increases the solute concentration. These competing mechanisms control evaporation at the latter stages of the droplet lifetime. At a certain point, due to continuous evaporation, the liquid mass completely depletes and evaporation stops. The droplet after complete desiccation consists only of salt crystals, probably encapsulating the viruses and rendering them inactive. If the SARS-CoV-2 virus would remain active within the salt crystal, also known as droplet nucleus or aerosol, Covid-19 could spread by aerosol transmission in addition to that by droplets. In this paper, we focus on infection spread exclusive by respiratory droplets since the role of aerosols is not clear for transmission of Covid-19.

## VI. RESULTS AND DISCUSSIONS

### A. Experimental validation

**FIG. 3.**
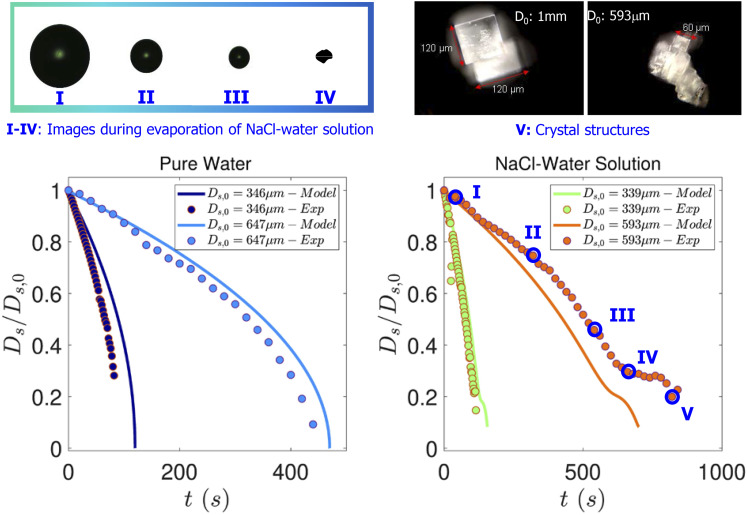
Instantaneous droplet images taken by a CCD camera (top left panel) and dark field micrograph of the final salt precipitate (top right panel). Comparison of experiments and simulations in the bottom left and right panels. Evolution of the normalized droplet diameter as a function of time for pure water (left panel) and the salt water solution droplet with 1% NaCl (right panel).

To validate the model, few targeted experiments were conducted to observe isolated levitated droplets evaporating in a fixed ambient condition. Particularly, the droplets with (1% w/w) NaCl solution vaporized to shrink to 30% of its initial diameter during the first stage of evaporation, as shown in Fig. 3. Hereafter, a plateau-like stage is approached due to increased solute accumulation near the droplet’s surface, which inhibits the diameter from shrinking rapidly. However, as shown in Fig. 3, shrinkage does occur (until *D*_*s*_/*D*_*s*,0_ ≈ 0.2) as the droplet undergoes a sol–gel transformation. The final shape of the precipitate is better observed from the micrographs presented in Fig. 3.

Figure 3 shows the final precipitate morphology for the desiccated droplets. The precipitates display a cuboid shaped crystalline formation, which is consistent with the structure of the NaCl crystal. The size and crystallite structure does show some variation, which could be linked with the initial size of the droplet. Only precipitates from larger droplets could be collected since smaller sized precipitates tend to de-stabilize and fly-off the levitator post-desiccation. While the precipitate from larger sized droplets tend to yield larger and less number of crystals, smaller droplets seem to degenerate into even smaller crystallites. However, this work does not investigate the dynamics of morphological changes of crystallization in levitated droplets.

Figure 3 also displays the comparison between results obtained from experiments and modeling. Experiments were performed with both pure water droplets as well as with droplets of 1% salt solutions. Experiments have been described in Sec. II. For the pure water cases shown in the left panel, simulation results follow the experiments rather closely. In the pure water case, classical *D*^2^ law behavior could be observed. For the salt water droplets, a deviation from the *D*^2^ law behavior occurs, and droplet evaporation is slowed. This is governed by Raoult’s law where the reduced vapor pressure *P*_*vap*_(*T*_*s*_, *χ*_1,*s*_) on the droplet surface results from the increasing salt concentration with time. The evaporation rate approaches zero at about *D*_*s*_/*D*_*s*,0_ for 0.3 (experiments) and 0.25 (simulations), respectively. However, the salt concentration attained at this stage exceeds the supersaturation *S* ≥ 1 required for onset of crystallization. Thus, the salt crystallizes, reducing its concentration and increasing *P*_*vap*_(*T*_*s*_, *χ*_1,*s*_) such that evaporation and water mass loss can proceed until nearly all the water has evaporated and only a piece of solid crystal as shown in Fig. 3 is left. It can be observed from Fig. 3 that in all cases, the final evaporation time is predicted within 15% of the experimental values. This suggests that despite the model being devoid of complexities (associated with inhomogeneities of temperature and solute mass fraction within the droplet and simple one step reaction to model the crystallization kinetics), it demonstrates reasonably good predictive capability. It is prudent to mention again that although we have done the analysis for the single isolated droplet, in reality, coughing or sneezing involves a whole gamut of droplet sizes in the form of a cloud.

Humans expel respiratory droplets while sneezing, coughing, or talking loudly. Such droplets have a size range from 5 *μ*m to 2000 *µ*m,^36^ while the dispersion could depend on the severity of the action. For example, while talking, an average human being will expel ∼600 droplets in a size range of 25 *µ*m–50 *µ*m, but this number goes upto ∼800 in the case of sneezing. For any given act (sneezing, coughing, or talking), the highest number of droplets fall in the range between 25 *µ*m and 50 *µ*m, while the smaller or larger droplets (than the above) are comparatively fewer in number. Nevertheless, the expelled volume of air contains a very small fraction of liquid droplets. To illustrate this point, the droplets are assumed to be in a uniform dispersion. The total volume of air expelled by a human being is estimated to be *V*_*g*_ ∼ 0.0005 m^3^. The total volume of liquid for a given mean droplet size is simply (*NV*_*D*,*s*,0_), where *N* is the total number of droplets of size *D*_*s*,0_ and *V*_*D*,*s*,0_ is the volume of such a droplet. The total volume occupied by droplets of different sizes in a given act of coughing or sneezing is $\sum_{i}N_{i}V_{D_{s,0},i}$. Thus, the volume fraction (*ϕ*) of liquid droplets during a given act is $\sum_{i}N_{i}V_{D_{s,0},i}/V_{g}$. Using size distribution, reported in Ref. 36, one can show that *ϕ* for sneezing, coughing, coughing with covered mouth, and talking loudly is 59 ppm, 549 ppm, 361 ppm, and 263 ppm, respectively. These numbers indicate that respiratory spray is rather sparse, implying that collective evaporation of droplet clusters may not be significant. This also justifies the modeling based on an isolated, contact free droplet.

### B. Ambience specific droplet lifetime

Next, we set out to use this model to predict the droplet lifetime characteristics over a wide range of ambient conditions. The droplet evaporation time *t*_*evap*_(*D*_*s*,0_) is calculated from the analysis presented in Secs. V A and V B. Indeed, the droplet evaporation competes with gravitational settling.^2^ The settling time *t*_*settle*_(*D*_*s*,0_) is calculated by accounting for the decreasing diameter using the equation for the Stokes settling velocity,

$$w=\left(\rho_{p}-\rho_{f}\right)gD_{s}^{2}/18\;\mu .$$ (23)

The settling time is estimated as that time by which the droplet gets out of the radius from which breathable air is collected—already defined as *σ*_*H*_/2 in Sec. III. Mathematically, *t*_*settle*_ is obtained by the following equation:

$$\int_{0}^{t_{settle}}wdt=\sigma_{H}/2.$$ (24)

Clearly, for any condition, while *t*_*evap*_ monotonically increases with *D*_*s*,0_, *t*_*settle*_ monotonically decreases with *D*_*s*,0_. In view of this, it is necessary to estimate the maximum time an exhaled droplet can remain within the collection volume without being evaporated or settled. Such a time can be estimated by defining a characteristic droplet lifetime *τ*, where

$$\tau =\min\left\{\;t_{evap}\;|\;t_{evap}\geq t_{settle}\forall D_{s,0}\right\}.$$ (25)

**FIG. 4.**
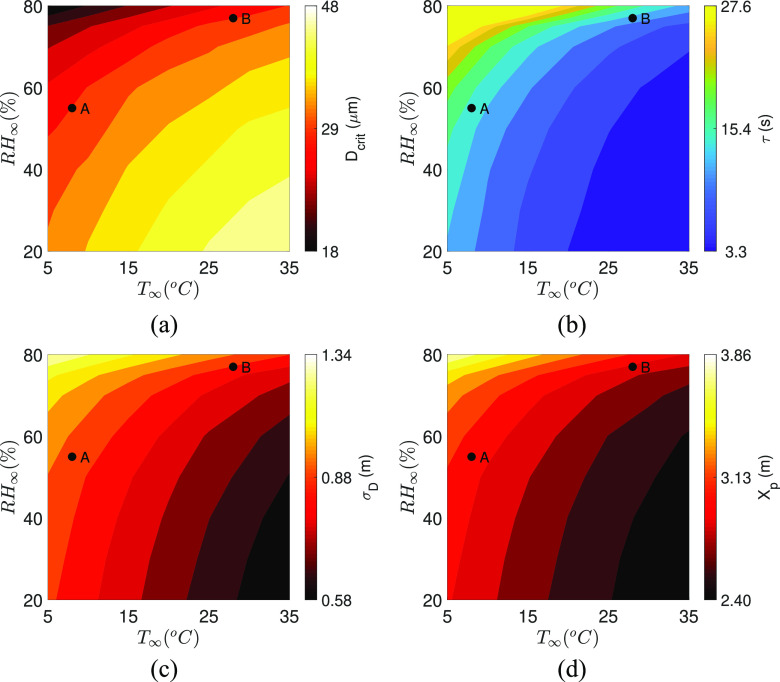
(a) *D*_*crit*_, (b) *τ*, (c) *σ*_*D*_, and (d) *X*_*p*_ as a function of *T*_∞_ and *RH*_∞_.The black dots A and B denote two typical conditions. Case A represents (*T*_∞_, *RH*_∞_) = (8, 55), while case B represents (*T*_∞_, *RH*_∞_) = (28, 77). Color bars have been clipped at reasonable values to show the respective variations over the wider region of interest.

*τ* is essentially the time where the two curves *t*_*evap*_, *t*_*evap*_ as a function of *D*_*s*,0_ intersect and represents the maximum time a liquid droplet of any size can exist before it is removed either by evaporation or gravity. For a given ambience specified by the ordered pair (*T*_∞_, *RH*_∞_), *D*_*s*,0_ corresponding to *τ* can be defined as *D*_*crit*_. Droplets with *D*_*s*,0_ > *D*_*crit*_ settle due to gravity, while *D*_*s*,0_ ≤ *D*_*crit*_ evaporate, earlier than the droplets with *D*_*s*,0_ = *D*_*crit*_. While droplets with *D*_*s*,0_ ≠ *D*_*crit*_ can certainly transmit the disease, those with *D*_*crit*_ establishes the boundaries in terms of the lifetime, cloud diameter, and maximum distance traversed. *D*_*crit*_ is dependent on ambient conditions, i.e., temperature and relative humidity. The distribution of *D*_*crit*_ over a wide range of relevant ambient conditions is shown in Fig. 4(a). Interestingly, at high *T*_∞_ and low *RH*_∞_, where the evaporation rate is very fast, even a large droplet rapidly shrinks before it can settle. By the same argument at low *T*_∞_ and high *RH*_∞_, a relatively smaller droplet cannot evaporate quickly; therefore, *t*_*evap*_ = *t*_*settle*_ is attained for smaller droplet sizes. This explains why we observe large *D*_*crit*_ at high *T*_∞_, low *RH*_∞_, and small *D*_*crit*_ at low *T*_∞_ and high *RH*_∞_. The evaporation time of the droplet of diameter *D*_*crit*_, which has been established as the characteristic lifetime *τ* of the droplet set, is shown in Fig. 4(b). Despite the different initial sizes, we find that *τ* is minimum at high *T*_∞_ and low *RH*_∞_ conditions, whereas it is maximum at low *T*_∞_ and high *RH*_∞_. At the same time, longer lifetime, i.e., large *τ*, allows the droplet cloud to travel a longer distance axially (*X*_*p*_) and disperse radially (*σ*_*D*_). Thus, *X*_*P*_ and *σ*_*D*_ are observed to be smaller (larger) for high (low) *T*_∞_ and low (high) *RH*_∞_ conditions, as shown in Figs. 4(c) and 4(d). This also shows how the minimum required “social distance” represented by *X*_*p*_ is not constant but depends on ambient conditions. Combining these results, it can be concluded that the size, lifetime, distance traveled, and the radial dispersion of the longest surviving droplet is not constant and is a strong function of ambient conditions. In particular, low temperature and high RH enhance the droplet lifetime significantly. Relative humidity strongly affects the droplet lifetime compared to temperature. An increase in droplet lifetime also implies that such droplets stay in the ambient for longer periods and hence travel longer distances as reflected by *X*_*p*_. This implies that such droplets can lead to higher infection propensities. The critical size, droplet lifetime, distance traveled, or size of the droplet cloud for any practical condition are readily obtainable from Figs. 4(a)–4(d), respectively.

**FIG. 5.**
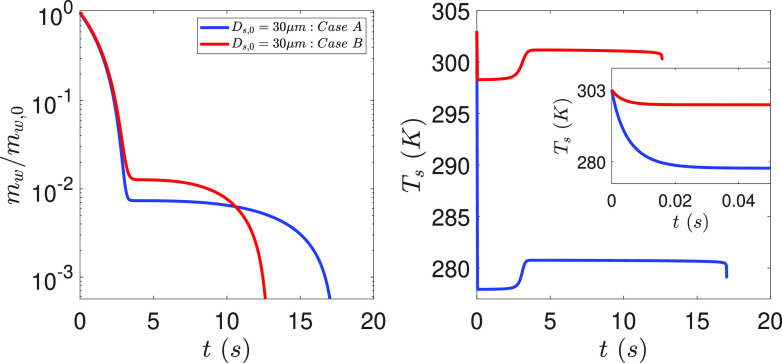
Evolution of the normalized mass of water in the droplet (left panel) and droplet temperature (right panel) as a function of time for cases A and B.

In Fig. 5, we look into the evolution of the normalized mass and temperature of the droplets for two cases named as case A and case B, also identified with black dots in Fig. 4. For case A, (*T*_∞_, *RH*_∞_) = (8, 55), while for case B, (*T*_∞_, *RH*_∞_) = (28, 77). The unit of *T*_∞_ is °C and that of RH is %. These conditions have been specifically chosen to loosely represent the spring weather in North America and South East Asia, respectively, for the early days of the Covid-19 pandemic at both these locations. Figure 5 clearly explains why *τ*_*A*_ > *τ*_*B*_. Indeed, higher RH at case B implies that the temporary hiatus in evaporation due to reduced vapor pressure is reached at a higher water mass load of the droplet than at the case A condition. In both cases, this occurs at about 3 s. However, the higher temperature in B results in faster crystallization kinetics due to the Arrhenius nature of the equation given by Eq. (19), which causes an eventual faster crystallization rate than at A. Figure 5 clearly shows that although the knee in the solvent depletion profiles are attained at the same time, it is the crystallization and simultaneous desiccation dynamics that governs the eventual difference in lifetime of the droplets at two conditions. It remains to be seen whether this result holds for a detailed crystallization reaction mechanism. The temperature evolution plots shown in the right panel of Fig. 5 also reveals how the droplet initially exhaled at 30 °C rapidly cools to the corresponding wet-bulb temperature to subsequently allow heat transfer into the droplet leading to evaporation. However, as the salt concentration reduces due to crystallization, the temperature rises subsequently above the corresponding wet-bulb limits.

### C. Calculated growth parameters and growth rates

**FIG. 6.**
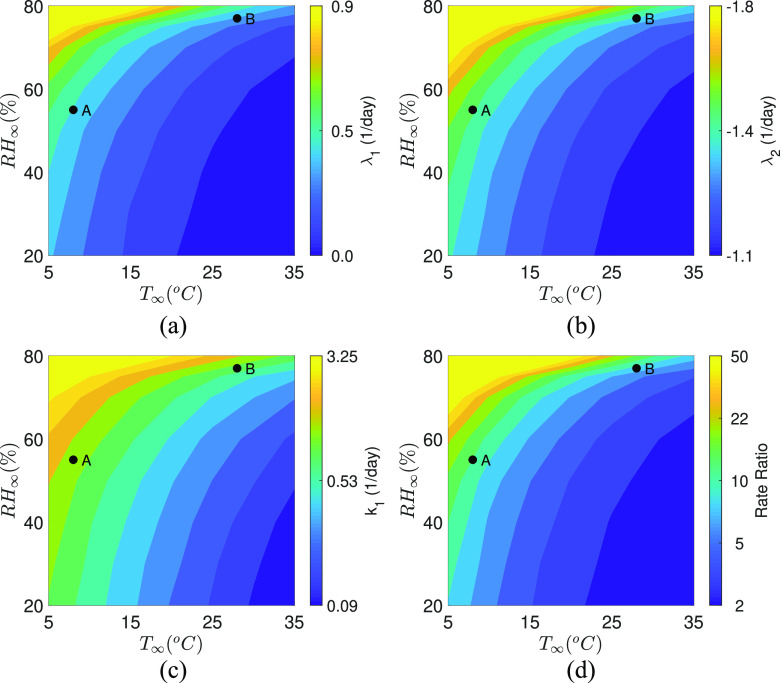
Contours of calculated eigenvalues (a) *λ*_1_ and (b) *λ*_2_ as a function of *T*_∞_ and *RH*_∞_. (c) Infection rate constant, *k*_1_, and (d) rate ratio over seven days as a function of *T*_∞_ and *RH*_∞_. Case A represents (*T*_∞_, *RH*_∞_) = (8, 55), while case B represents (*T*_∞_, *RH*_∞_) = (28, 77). The rate ratio for A and B are 16.60 and 10.33, respectively. Color bars have been clipped at reasonable values to show the respective variations over the wider region of interest.

With the droplet lifetime available over a wide range of conditions, the corresponding infection rate constant and eigenvalues given by Eqs. (5) and (6) could be evaluated. Just to recapitulate, *τ* determines the infection rate constant *k*_1_ by Eq. (14). In turn, *k*_1_ affects the exponents of the time dependent infection equation [Eq. (7)], the growth parameters—eigenvalues *λ*_1_, *λ*_2_ through Eq. (8). The contours of *λ*_1_, *λ*_2_, and *k*_1_ as a function of *T*_∞_ and *RH*_∞_ are shown in Figs. 6(a)–6(c), respectively. The direct correspondence between *τ* and *k*_1_ is immediately apparent upon comparing the respective Figs. 4(b) and 6(c). The infection rate constant is highest at low *T*_∞_ and high *RH*_∞_ where the droplet evaporation is slowed due to the slow mass loss rate and enhanced crystallization time. On the other hand, faster droplet evaporation leads to small infection rate constant values at high *T*_∞_ and low *RH*_∞_. The temperature and relative humidity dependency of the eigenvalues *λ*_1_ and *λ*_2_ are shown in Figs. 6(a) and 6(b), respectively. The direct correspondence of Figs. 6(a) and 6(b) with Fig. 6(c) and Fig. 4 are established through $\lambda_{1,2}=-0.54\pm \sqrt{0.22+k_{1}}$ given by Eq. (8). It should, however, be noted that due to the inherent negative sign of *λ*_2_, its influence on determining the growth rate of the infected population is rather limited. It is *λ*_1_ that primarily drives the growth of the infected population as apparent from Eq. (7). From Fig. 6(a), we observe that for a fixed *T*_∞_, *λ*_1_ increases with *RH*_∞_, while for a fixed *RH*_∞_, *λ*_1_ decreases with *T*_∞_. Furthermore, we observe that the iso-*λ*_1_ contour lines bend and converge at *RH*_∞_ > 75%. This means that for *RH*_∞_ > 75%, large *λ*_1_ > 0.4 is expected over a wider range of temperatures between 5 °C < *T*_∞_ < 20 °C. This is a manifestation of the greatly reduced evaporation potential—the difference between water vapor concentration on the droplet surface and in the ambient at high *RH*_∞_ conditions. This is further reflected in the dramatic difference in the rate ratio—the ratio of the cumulative number of positive cases on a particular day to the cumulative number of positive cases seven days before: *N*_*P*_/*N*_*P*,0_ in Fig. 6(d). This figure has been arrived at by assuming the local population density to be 10 000 km^−2^. Furthermore, we calculate *t*_*c*_ in Eq. (14) as *t*_*c*_ = 3600 × 24/*N*_exp_. *N*_exp_ is the number of infecting expiratory events per person per day and is assumed to be 3 based on the coughing frequency of 0–16 in normal subjects.^37^ We find that purely based on ambient conditions, implying all other factors have been held constant, the rate ratio can be different by an order of magnitude between (*T*_∞_, *RH*_∞_) = (5, 75) vs (35, 20). Practically, such a contrast might be less apparent in real data in which other important factors such as population density, social enforcement, travel patterns, and susceptible supply^38^ exert significant influence.

## VII. SUMMARY

**FIG. 7.**
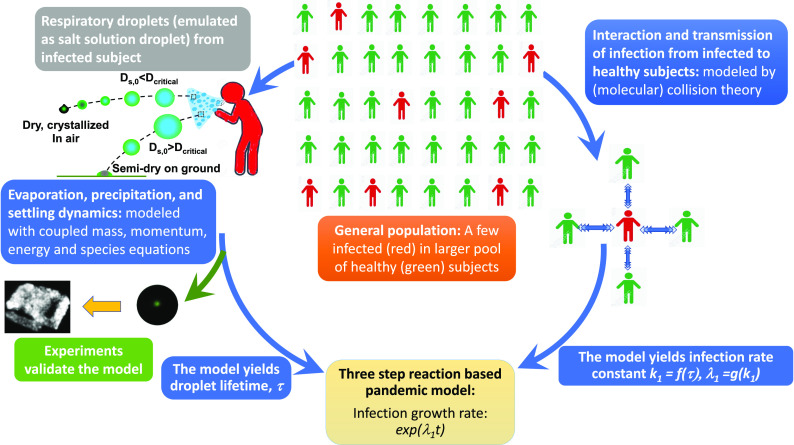
Flow-diagram outlining the interconnections of the model developed.

Respiratory flow ejected by human beings consists of a polydisperse collection of droplets. In this paper, we have presented a model for the early phases of a Covid-19 like pandemic based on the aerodynamics and evaporation characteristics of respiratory droplets. The model and its inter-dependencies on the different physical principles/sub-models are summarized in Fig. 7. To our knowledge, this is the first model that utilizes the structure of a chemical reaction mechanism to connect the pandemic evolution equations with respiratory droplet lifetime by first principles modeling of the reaction rate constant. However, it must be recognized that the model assumes conditions where transmission occurs solely due to inhalation of infected respiratory droplets alongside many other simplifying assumptions. The evolution of the droplets is characterized by a complex interaction of aerodynamics, evaporation thermodynamics, and crystallization kinetics. As such, after being ejected, smaller droplets attain the wet-bulb temperature corresponding to the local ambience and begin to evaporate. However, due to the presence of dissolved salt, the evaporation stops when the size of the droplet reaches about 20%–30% of the initial diameter, but now, the droplet salt concentration has increased to levels that trigger onset of crystallization. Of course, these processes compete with settling—the process by which larger droplets fall away before they can evaporate. The smaller of the two, complete evaporation time and settling time, thus dictates the droplet lifetime *τ*. The infection rate constant derived using collision theory of reaction rates is shown to be a function of the respiratory droplet lifetime (*τ*), where *τ* is sensitive to ambient conditions. While the infection rate constant in reality is dependent on numerous parameters, the present approach allows us to compute its exclusive dependence on ambient conditions through respiratory droplet modeling. We find that the respiratory droplets exclusively contribute to the infection growth parameters and infection growth rate, which decrease with ambient temperature and increase with relative humidity. As such, the model could be used for providing fundamental insights into the role of respiratory droplets in Covid-19 type viral disease spread. Furthermore, the model could be used, with extreme caution and in cognizance of its limitations, toward estimating the risk potential of infection spread by droplet transmission for specific ambient conditions of interest from purely physics based calculations.

## DATA AVAILABILITY

The data that support the findings of this study are available from the corresponding authors upon reasonable request.
